# ABCG1 and ABCG4 Suppress γ-Secretase Activity and Amyloid β Production

**DOI:** 10.1371/journal.pone.0155400

**Published:** 2016-05-19

**Authors:** Osamu Sano, Maki Tsujita, Yuji Shimizu, Reiko Kato, Aya Kobayashi, Noriyuki Kioka, Alan T. Remaley, Makoto Michikawa, Kazumitsu Ueda, Michinori Matsuo

**Affiliations:** 1 Laboratory of Cellular Biochemistry, Division of Applied Life Sciences, Kyoto University Graduate School of Agriculture, Kyoto, 606–8502, Japan; 2 Biochemistry, Graduate School of Medical Sciences, Nagoya City University, Nagoya, 467–8601, Japan; 3 Lipoprotein Metabolism Section, NHLBI, National Institutes of Health, Bethesda, MD, 20892–1508, United States of America; 4 iCeMS, Kyoto University, Kyoto, 606–8502, Japan; 5 Department of Food and Nutrition, Faculty of Home Economics, Kyoto Women’s University, Kyoto, 605–8501, Japan; Sungkyunkwan University, REPUBLIC OF KOREA

## Abstract

ATP-binding cassette G1 (ABCG1) and ABCG4, expressed in neurons and glia in the central nervous system, mediate cholesterol efflux to lipid acceptors. The relationship between cholesterol level in the central nervous system and Alzheimer’s disease has been reported. In this study, we examined the effects of ABCG1 and ABCG4 on amyloid precursor protein (APP) processing, the product of which, amyloid β (Aβ), is involved in the pathogenesis of Alzheimer’s disease. Expression of ABCG1 or ABCG4 in human embryonic kidney 293 cells that stably expressed Swedish-type mutant APP increased cellular and cell surface APP levels. Products of cleavage from APP by α-secretase and by β-secretase also increased. The levels of secreted Aβ, however, decreased in the presence of ABCG1 and ABCG4, but not ABCG4-KM, a nonfunctional Walker-A lysine mutant. In contrast, secreted Aβ levels increased in differentiated SH-SY5Y neuron-like cells in which ABCG1 and ABCG4 were suppressed. Furthermore, Aβ42 peptide in the cerebrospinal fluid from Abcg1 null mice significantly increased compared to the wild type mice. To examine the underlying mechanism, we analyzed the activity and distribution of γ-secretase. ABCG1 and ABCG4 suppressed γ-secretase activity and disturbed γ-secretase localization in the raft domains where γ-secretase functions. These results suggest that ABCG1 and ABCG4 alter the distribution of γ-secretase on the plasma membrane, leading to the decreased γ-secretase activity and suppressed Aβ secretion. ABCG1 and ABCG4 may inhibit the development of Alzheimer’s disease and can be targets for the treatment of Alzheimer’s disease.

## Introduction

Alzheimer’s disease is characterized by extracellular senile plaques in brain tissues [1]. Amyloid β (Aβ), a major component of the senile plaques, plays a crucial role in the pathogenesis of Alzheimer’s disease. This peptide can be 40 (Aβ40) or 42 (Aβ42) amino acids in length, after cleavage of amyloid precursor protein (APP). The precursor is cleaved by α-secretase to produce secreted APPα (sAPPα) and carboxy-terminal fragment α (CTFα) or β-secretase to produce sAPPβ and CTFβ, which is further cleaved by γ-secretase to produce Aβ and CTFγ.

Although the brain represents 3% of the average body mass, it contains 25% of the cholesterol in the body. Cholesterol levels in the brain are regulated independently of peripheral systems because cholesterol cannot cross the blood−brain barrier [2]. Cholesterol in the central nervous system (CNS) is supplied by synthesis, and excess cholesterol is converted to 24-hydroxycholesterol by CYP46A1. High levels of cholesterol are found in myelin (oligodendrocytes) in the CNS, although neurons and other glial cells also contain cholesterol. Cholesterol and apolipoprotein E (apoE) are synthesized in astrocytes, leading to the formation of apoE-containing lipoprotein (LpE), a high-density lipoprotein (HDL)-like particle that is provided to neurons and used as a component of cellular membranes or to support synaptogenesis [3] and axonal extension [4]. The correlation between cholesterol level in the brain and Alzheimer’s disease has been reported. The addition of statin or methyl-β-cyclodextrin (MβCD) reduces the Aβ formation in rat hippocampal neurons [5]. The administration of statin reduces Aβ levels in rat neurons and guinea pigs [6], whereas hypercholesterolemia accelerates Aβ accumulation in Alzheimer’s disease model mice [7, 8]. Cholesterol is accumulated in senile plaques from Alzheimer’s disease patients and Alzheimer’s disease model mice [9]. There are three major alleles of human apoE: apoE2, apoE3, and apoE4. ApoE4 is a strong risk factor for Alzheimer’s disease, whereas apoE2 is associated with lower risk for the disease [10]. This may reflect allele-specific differences in cholesterol delivery to neurons by LpE.

Other studies have examined the relationships between cholesterol levels and secretase activities. Depletion of cholesterol reduced β-secretase and γ-secretase activities, leading to decreased production of Aβ, suggesting that β-secretase and γ-secretase function in lipid raft domains [11, 12]. Of note, Aβ aggregation occurs in these domains [13].

ATP-binding cassette G1 (ABCG1) and ABCG4 are half-type ABC proteins that belong to the G subfamily of the ABC superfamily. ABCG1 and ABCG4 form functional homodimers [14], and may form heterodimers [15]. ABCG1 and ABCG4 mediate the efflux of cholesterol to HDL in human embryonic kidney (HEK) cells and baby hamster kidney cells [16–18]. ABCG1 and ABCG4 are highly expressed in the CNS and mediate the efflux of cholesterol to LpE [19–21]. ABCG1 is expressed in both neurons and astrocytes, whereas ABCG4 has been detected in neurons and astrocytes [22], as well as microglia from patients with Alzheimer’s disease [23]. Both ABCG1 and ABCG4 are thought to be involved in sterol homeostasis in the body. A lack of Abcg1 caused significant accumulation of neutral lipids in macrophages when the mutant mice were fed a high-fat, high-cholesterol diet [24]. Furthermore, mice lacking Abcg1 showed severe age-dependent pulmonary lipidosis [25]. On the other hand, overexpression of ABCG1 protected murine tissues from lipid accumulation [24]. The absence of Abcg4 in mice did not affect brain levels of cholesterol, whereas brain levels of lathosterol increased [26]. Moreover, ABCG4 mediated desmosterol efflux [26]. Double-knockout mice lacking Abcg1 and Abcg4 showed significantly increased desmosterol levels in the brain, suggesting that ABCG1 and ABCG4 have overlapping functions. Bojanic *et al*. reported that Abcg4^−/−^ mice had a general deficit in associated fear memory, suggesting that ABCG4 contributes to neuronal plasticity via moving lipids [27]. Furthermore, Abcg4^−/−^ mice showed increased proliferation of megakaryocyte progenitor cells, indicating that ABCG4 regulates proliferation of cells [28].

It has been reported that ABCA1, which is a member of the A subfamily and mediates the efflux of cholesterol and phosphatidylcholine to lipid-free apoA-I or apoE [14, 21, 29], reduced Aβ secretion from cells [30, 31]. Aβ deposition increased in the absence of Abca1 in mouse models of Alzheimer’s disease [32–34] and decreased by the overexpression of ABCA1 [35], although another study showed that the absence of Abca1 did not affect Aβ levels in mice [36]. Because, like ABCA1, ABCG1 removes excess cholesterol from cells and is expressed in the CNS, we investigated the effects of the expression of ABCG1 on Aβ secretion. Furthermore, ABCG1 has been reported to be associated with a risk for Alzheimer’s disease [37]. Kim *et al*. demonstrated that expression of ABCG1 in Chinese hamster ovary cells that stably expressed APP suppressed Aβ generation [21]. In contrast, Tansley *et al*. showed that ABCG1 expression in HEK cells expressing a Swedish-type APP mutant increased Aβ secretion [38]. Aβ levels in both ABCG1-overexpressing and Abcg1-deficient mice, however, were unchanged [39]. The reason for these discrepancies is unclear, as is the mechanism underlying altered APP processing in response to expression of ABCG1. Furthermore, no published studies have addressed on whether ABCG4 also affects APP processing.

We previously reported that ABCG1 mediates the efflux of sphingomyelin, phosphatidylcholine, and cholesterol from cells [14]. Furthermore, cholesterol and sphingomyelin synergistically stimulate ATP hydrolysis rate of purified ABCG1 [40]. Sphingomyelin and cholesterol form ordered microdomains (raft domains) in the plasma membrane. ABCG1 increases the cholesterol accessible to cholesterol oxidase [41], suggesting that ABCG1 decreases the raft domains and concomitantly increases the non-raft domains. We have demonstrated that the expression of ABCG1 and ABCG4 decreased the amounts of caveolin-1 in the raft domains [42], suggesting that ABCG1 and ABCG4 disturb the raft domains on the plasma membrane. Because γ-secretase functions in the raft domains [11, 43], we hypothesized that ABCG1 and ABCG4 affect γ-secretase activity and Aβ secretion.

To assess the effects of ABCG4 on APP processing and to elucidate how ABCG1 alters APP processing, we examined APP processing and Aβ secretion in cells expressing ABCG1 or ABCG4. We demonstrated that expression of ABCG1 or ABCG4 changed the distribution of γ-secretase on the plasma membrane and suppressed Aβ secretion.

## Materials and Methods

### Materials

Mouse monoclonal anti-APP antibody was purchased from Covance (Princeton, NJ). Rat polyclonal anti-presenilin-1 antibody was obtained from Chemicon (Temecula, CA). Rabbit polyclonal anti-nicastrin antibody was obtained from Sigma-Aldrich (St. Louis, MO). Rabbit polyclonal anti-ABCG1 antibody was obtained from Santa Cruz (Dallas, Texas). HDL was acquired from Calbiochem (San Diego, CA). Other chemicals were purchased from Sigma-Aldrich, GE Healthcare (Little Chalfont, UK), Cayman Chemical (Ann Arbor, MI), Wako Pure Chemical Industries (Osaka, Japan), and Nacalai Tesque (Kyoto, Japan).

### Experimental animals

Abcg1 null mouse with C57/BL6 background were purchased from Deltagen, Inc (San Mateo, CA). The C57BL/6 mice were obtained from a local supplier. The experiment animals were kept at the Center for Experimental Animal Science, Nagoya City University Graduate School of Medical Sciences Maintained with MF standard feeding chow (Oriental Yeast Co., Tokyo, Japan) in 25°C room with 12 h light–dark cycles. The experimental procedure was approved by Animal Welfare Committee of Nagoya City University Graduate School of Medical Sciences according to the institutional guidelines (approval number H13-112).

### Constructs

*APP* cDNA (GenBank accession number NM_000484) was amplified from a human whole brain cDNA library by PCR using two primers (forward, 5’-aagcttgtgatgctgcccggtttggc-3’; reverse, 5’-tctagactagttctgcatctgctcaaag-3’). The cloned cDNA was subcloned into the *Hind*III-*Xba*I sites of pCR2.1 (Invitrogen, Carlsbad, CA). A Swedish mutation (K594N and M595L) was introduced by changing g1782 and a1783 to c using a QuikChange II Site-Directed Mutagenesis Kit (Stratagene, La Jolla, CA) according to the manufacturer’s instructions. Human Swedish mutant *APP* cDNA was inserted into the *Hind*III-*Xba*I sites of pcDNA3.1(+)Hygro (Invitrogen) to create the pcDNA3.1Hygro(+)/APPsw expression vector. Human *ABCG4* cDNA [44] was inserted into the *Not*I and *EcoR*I sites of pcDNA3.1/Hygro(+) to create pcDNA3.1Hygro(+)/ABCG4. A Walker A lysine codon was changed to a methionine codon using a QuikChange II Site-Directed Mutagenesis Kit to create the pcDNA3.1Hygro(+)/ABCG4-KM expression vector.

### Cell culture

HEK293 cells and SH-SY5Y cells (American Type Culture Collection) were grown in Dulbecco’s modified Eagle’s medium (DMEM) supplemented with 10% (v/v) fetal bovine serum in 5% CO_2_ at 37°C.

### Establishment of a stable transformant of APPsw

HEK293 cells were transfected with pcDNA3.1Hygro(+)/APPsw using Lipofectamine (Invitrogen) according to the manufacturer’s instructions. Cells were selected with hygromycin B for 5 days and single colonies were isolated.

### Transfection of ABCG1 and ABCG4

HEK293 or HEK293 cells stably expressing APPsw (HEK/APPsw) were transfected with pcDNA3.1(+)/ABCG1 [14], pcDNA3.1(+)/ABCG1-KM, pcDNA3.1Hygro(+)/ABCG4, or pcDNA3.1Hygro(+)ABCG4-KM using Lipofectamine according to the manufacturer’s instructions.

### Reduction of ABCG1 or ABCG4 expression by RNA silencing

Control small interfering RNA (siRNA; Stealth RNAi Negative Control Medium GC Duplex #2); two siRNAs targeting human ABCG1, that is, ABCG1#1 (ABCG1-HSS145233) and ABCG1#2 (ABCG1-HSS-190466); and two siRNAs targeting human ABCG4, ABCG4#1 (ABCG4-HSS127417) and ABCG4#2 (ABCG4-HSS-127418) were purchased from Invitrogen. SH-SY5Y cells were transfected with 10 nM siRNAs using Lipofectamine RNAiMAX (Invitrogen) [45]. For differentiation of SH-SY5Y cells, 10 μM all-*trans* retinoic acid was added to the culture medium and the cells were cultured for 3 days. After differentiation, SH-SY5Y cells were incubated for 16 h in DMEM containing 0.02% bovine serum albumin (BSA) in the presence or absence of 5 μM TO901317, a synthetic ligand of liver X receptor (LXR) and 5 μM 9-*cis* retinoic acid (RA), a ligand of retinoid X receptor (RXR).

### OptiPrep gradient ultracentrifugation

Cells were washed with phosphate-buffered saline and collected. The cells were resuspended in TNE buffer (25 mM Tris-Cl (pH 7.4), 5 mM EDTA, and 150 mM NaCl) containing 100 μg/ml (*p*-amidinophenyl)methanesulfonyl fluoride, 2 μg/ml leupeptin, and 2 μg/ml aprotinin, and passed through a 26-G needle 10 times. Broken cells were centrifuged at 3,000 × *g* for 5 min and TNE buffer containing 2% Triton X-100 was added to achieve a final Triton X-100 concentration of 1%. Samples were then incubated for 15 min on ice. Raft domains were isolated using a discontinuous OptiPrep gradient consisting of the following layers: 400 μl of 35% opti and lysates, 1,600 μl of 30% opti/TNE buffer, and 200 μl of TNE buffer [46]. The gradient was centrifuged in a TLS55 rotor (Beckman Coulter, Brea, CA) at 4°C for 4 h at 200,000 × *g*. After centrifugation, ten 200-μl fractions were collected from the top of the tube and proteins were precipitated using acetone. The pellet was resuspended in SDS sample buffer and subjected to immunoblotting.

### Western blotting

Cells were washed with phosphate-buffered saline and lysed in lysis buffer (50 mM Tris-Cl (pH 7.5), 150 mM NaCl, and 1% Triton X-100) containing 100 μg/ml (*p*-amidinophenyl)methanesulfonyl fluoride, 2 μg/ml leupeptin, and 2 μg/ml aprotinin. Samples were electrophoresed on a 5−20% or 10% SDS-polyacrylamide gel and detected with antibodies.

### Collection of mouse cerebrospinal fluid

Each mouse was exsanguinated under anesthetic maintenance to reduce vascular pressure. Cerebrospinal fluid (CSF) was harvest from cisterna magna, as described previously [47, 48]. In short, using a dissecting microscope, the cisterna magna carefully exposed. The arachnoid membrane was punctured with a 29G insulin syringe (TERUMO, Tokyo, Japan) and CSF was collected from the cistern compartment. The CSF was then transferred into microtubes and immediately frozen on dry ice, and stored at -80°C until the analysis.

### Measurement of Aβ levels

Media were collected and Aβ40 and Aβ42 levels were measured using a Human β Amyloid ELISA Kit (Wako Pure Chemical Industries) according to the manufacturer’s instructions. For mouse CSF Aβ detection, Human/Rat β Amyloid (40) ELISA kit Wako II and (42) ELISA Kit Wako, High-Sensitive were used.

### Measurement of γ-secretase activity

Cells were collected and γ-secretase activity was measured using a γ-secretase activity kit (R&D Systems, Minneapolis, MN) according to the manufacturer’s instructions.

### Statistical analysis

Values are presented as means ± SD. Statistical significance among groups was determined using ANOVA followed by Dunnett’s test. *P* < 0.05 was considered statistically significant.

## Results

### ABCG1 and ABCG4 increase APP levels

**Fig 1:**
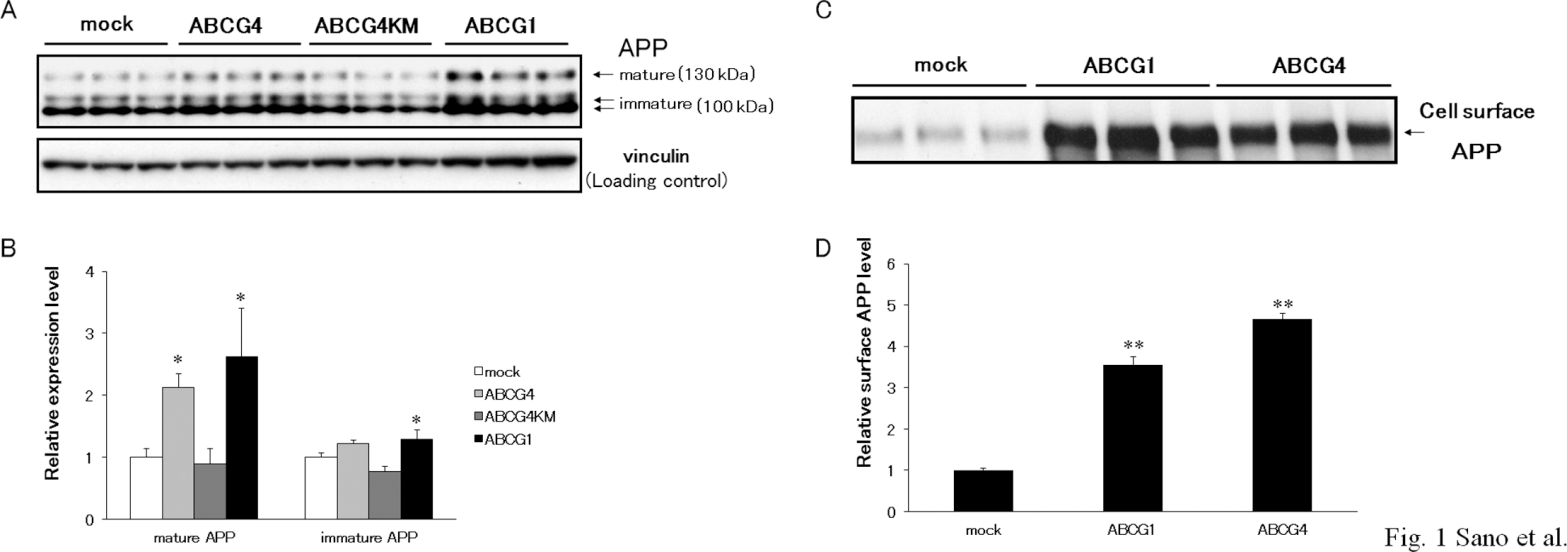
ABCG1 and ABCG4 increased cellular and surface levels of APP. (A) HEK/APPsw cells were transiently transfected with ABCG4, ABCG4-KM, or ABCG1, or mock-transfected. Twenty-four hours after transfection, cells were collected and cellular APP was detected by immunoblotting. Vinculin was used as a loading control. (B) The amounts of mature APP and immature APP detected by immunoblotting were analyzed. The data represent the expression levels of APP normalized by vinculin relative to that in mock-transfected cells. Values are represented with the SD. * *P* < 0.05, significantly different from mock-transfected cells. (C) HEK/APPsw cells, transiently transfected with ABCG1 or ABCG4, or mock-tansfected, were treated with sulfo-NHS-biotin followed by precipitation of biotinylated surface proteins using streptavidin agarose beads. Cell surface APP was detected by immunoblotting. All experiments were carried out in triplicate. (D) The amounts of cell surface APP detected by immunoblotting were analyzed. The data represent the expression levels of surface APP normalized by total APP proteins relative to that in mock-transfected cells. Values are represented with the SD. ** *P* < 0.01, significantly different from mock-transfected cells.

**Fig 2:**
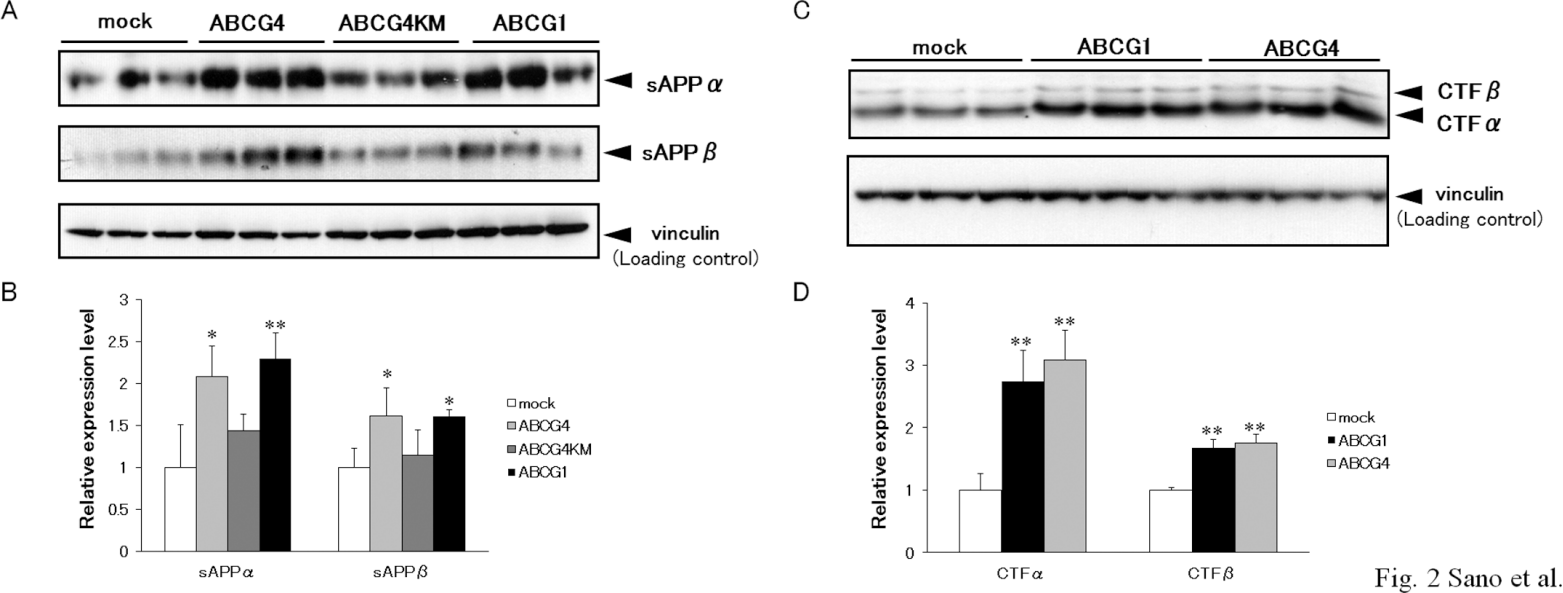
ABCG1 and ABCG4 promoted the release of sAPPα and sAPPβ, and increased the levels of CTFα and CTFβ. HEK/APPsw cells were transfected with ABCG4, ABCG4-KM, or ABCG1, or mock-transfected. Twenty-four hours after transfection, cells were incubated in DMEM containing 0.02% bovine serum albumin (BSA) for 24 h. (A) Media were collected, and sAPPα and sAPPβ levels were evaluated on immunoblots. (B) The amounts of sAPPα and sAPPβ detected by immunoblotting were analyzed. The data represent the expression levels of sAPPα and sAPPβ normalized by vinculin relative to that in mock-transfected cells. Values are represented with the SD. * *P* < 0.05; ** *P* < 0.01 significantly different from mock-transfected cells. (C) Cells were collected, and cellular CTFα and CTFβ were detected by immunoblotting. (D) The amounts of CTFα and CTFβ detected by immunoblotting were analyzed. The data represent the expression levels of CTF normalized by vinculin relative to that in mock-transfected cells. Values are represented with the SD. ** *P* < 0.01 significantly different from mock-transfected cells. All experiments were carried out in triplicate.

The effects of ABCG4 have not been investigated, although the processing of APP in the presence of ABCG1 has been examined in the previous studies [21, 38]. To examine APP processing, ABCG1, ABCG4, and ABCG4-KM, which is a Walker A lysine mutant of ABCG4, were transiently expressed in HEK/APPsw cells. As reported by Kim *et al*. [21], cellular APP levels increased in response to expression of ABCG1 (Fig 1A and 1B). Two bands (130 and 100 kDa) were detected on immunoblots. The 130-kDa band was insensitive to endoglycosidase H but was sensitive to PNGase F, whereas the 100-kDa band was sensitive to both endoglycosidase H and PNGase F (data not shown), demonstrating that the bands were mature and immature forms of APP, respectively [49, 50]. Levels of mature and immature APP increased in cells expressing ABCG1 compared with mock-transfected cells. Expression of ABCG4 also increased the levels of mature APP, whereas expression of non-functional ABCG4-KM did not. Because APP is cleaved at the plasma membrane and/or in the endosome [12, 51, 52], cell surface APP levels were investigated in a biotinylation assay. ABCG1 and ABCG4 markedly increased amounts of APP in the plasma membrane (Fig 1C and 1D). To examine if this effect is specific to APP or universal to other proteins, we checked expression levels of another membrane protein, sodium potassium ATPase (S1A and S1B Fig). Neither ABCG1 nor ABCG4 affected expression of sodium potassium ATPase, suggesting that the increased expression is specific to APP. It is unlikely that transcription of *APP* was increased because cells stably expressing APP were used in this experiment. Thus, we speculated that the expression of ABCG1 and ABCG4 suppressed degradation of APP. Indeed, the half-life of APP increased in the presence of ABCG1 or ABCG4 (S2 Fig). When protein synthesis was inhibited in mock-transfected cells, less than 20% of the mature APP remained after 1 h. When ABCG1 or ABCG4 was expressed in the cells, about 40% of the mature APP remained after treatment with cycloheximide for 1 h. Next, we examined products of APP cleavage: sAPPα, sAPPβ, CTFα, and CTFβ. Levels of sAPPα and sAPPβ were higher in cells expressing ABCG1 or ABCG4 than in mock-transfected cells (Fig 2A and 2B). Levels of sAPPα and sAPPβ in cells expressing ABCG4-KM were similar with those in mock-transfected cells. Furthermore, expression of ABCG1 and ABCG4 in HEK/APPsw cells increased both CTFα and CTFβ (Fig 2C and 2D). These findings suggest that increased APP levels enhanced the production of sAPPα, sAPPβ, CTFα, and CTFβ by α-secretase and β-secretase.

### Secreted Aβ from cells expressing ABCG1 or ABCG4 is reduced

**Fig 3:**
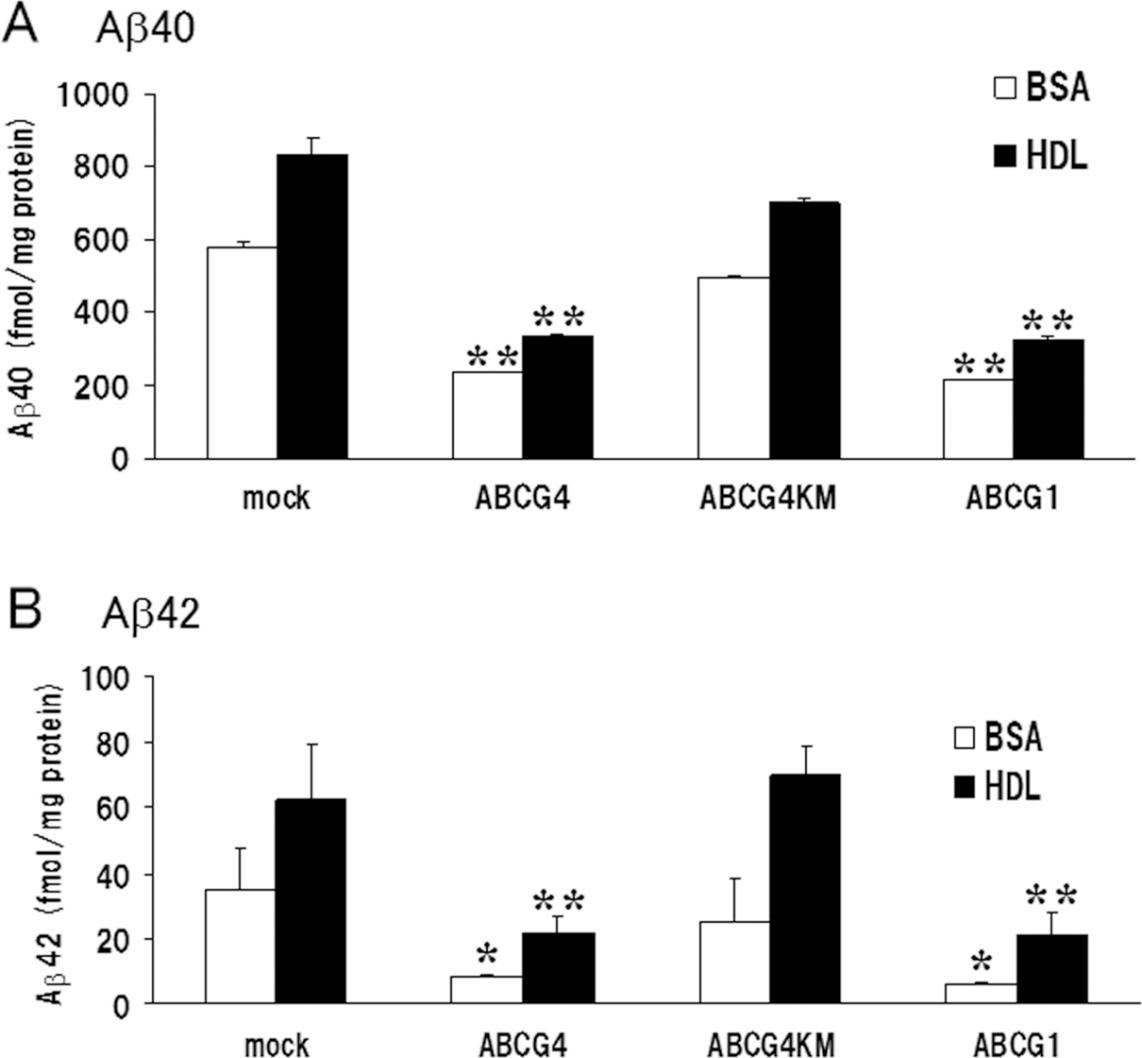
ABCG1 and ABCG4 suppressed Aβ secretion. HEK/APPsw cells were transiently transfected with ABCG4, ABCG4-KM, or ABCG1, or mock-transfected. Twenty-four hours after transfection, cells were incubated in DMEM containing 0.02% BSA (open bars) or 0.02% BSA and 20 μg/ml HDL (filled bars) for 24 h. Media were collected, and Aβ40 (A) and Aβ42 (B) levels were measured using an enzyme-linked immunosorbent assay. Values from three experiments were normalized based on total cellular protein, and means are represented with the SD. * *P* < 0.05; ** *P* < 0.01 compared with mock-transfected cells.

Accumulation of Aβ is one of the characteristic features found in patients with Alzheimer’s disease. To investigate whether Aβ levels increased together with the elevation of CTFβ levels, we measured amounts of Aβ secreted from cells expressing ABCG1 or ABCG4. We have shown that the presence of HDL in the medium enhances the efflux of lipids mediated by ABCG1 compared with that in the presence of only BSA [14]. Moreover, ABCG4 mediates efflux of cholesterol in an HDL-dependent manner [26]. To examine whether lipid efflux affected Aβ secretion, the amounts of secreted Aβ were measured in the presence or absence of HDL. Expression of ABCG1 in HEK/APPsw cells significantly suppressed the secretion of Aβ40 into the medium compared with levels observed for cells subjected to mock transfection (Fig 3A), which is consistent with the study by Kim *et al*. [21]. Aβ40 secretion was suppressed even in the absence of HDL in the medium, suggesting that lipid efflux is not necessary for the suppression of Aβ40 secretion. In the presence of HDL, more Aβ was secreted from cells than in its absence. This suggests that HDL in the medium rather enhanced Aβ40 secretion. Expression of ABCG4 also suppressed the secretion of Aβ40, whereas expression of ABCG4-KM mutant did not affect it. Similar results were observed for Aβ42 (Fig 3B). ABCG1 and ABCG4 suppressed Aβ42 secretion irrespective of the presence of HDL, whereas ABCG4-KM did not affect the secretion of Aβ42. These findings indicate that, like ABCG1, ABCG4 decreased the production of Aβ.

### ABCG1 and ABCG4 reduce γ-secretase activity

**Fig 4:**
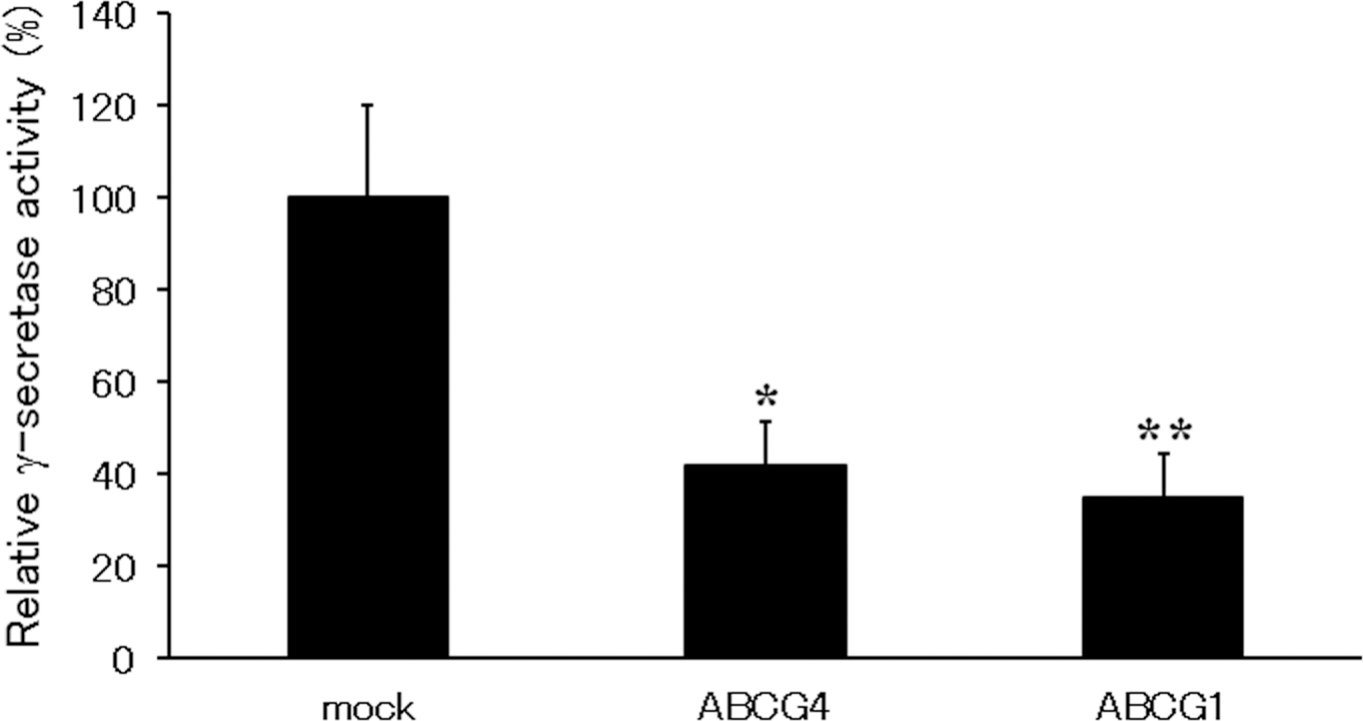
ABCG1 and ABCG4 suppressed γ-secretase activity. HEK/APPsw cells were transfected with ABCG4 or ABCG1, or mock-transfected. Twenty-four hours after transfection, cells were incubated in DMEM containing 0.02% BSA for 24 h. The activity of endogenous γ-secretase was determined using a γ-secretase assay kit. Experiments were performed in triplicate and the activities relative to those observed in mock-transfected cells are represented as means with the SD. * *P* < 0.05; ** *P* < 0.01 compared with mock-transfected cells.

Fig 3 suggests that the production of Aβ by γ-secretase is reduced by ABCG1 and ABCG4, although the amounts of the γ-secretase substrate CTFβ increased (Fig 2). We speculated that γ-secretase activity was reduced by the expression of ABCG1 or ABCG4. To examine this, we measured γ-secretase activity. The activities of γ-secretase in cells expressing ABCG1 or ABCG4 were approximately 30% or 40% of that in mock-transfected cells, respectively (Fig 4), suggesting that the expression of either ABCG1 or ABCG4 reduces the γ-secretase activity.

### Distribution of γ-secretase to raft domains is disturbed

**Fig 5:**
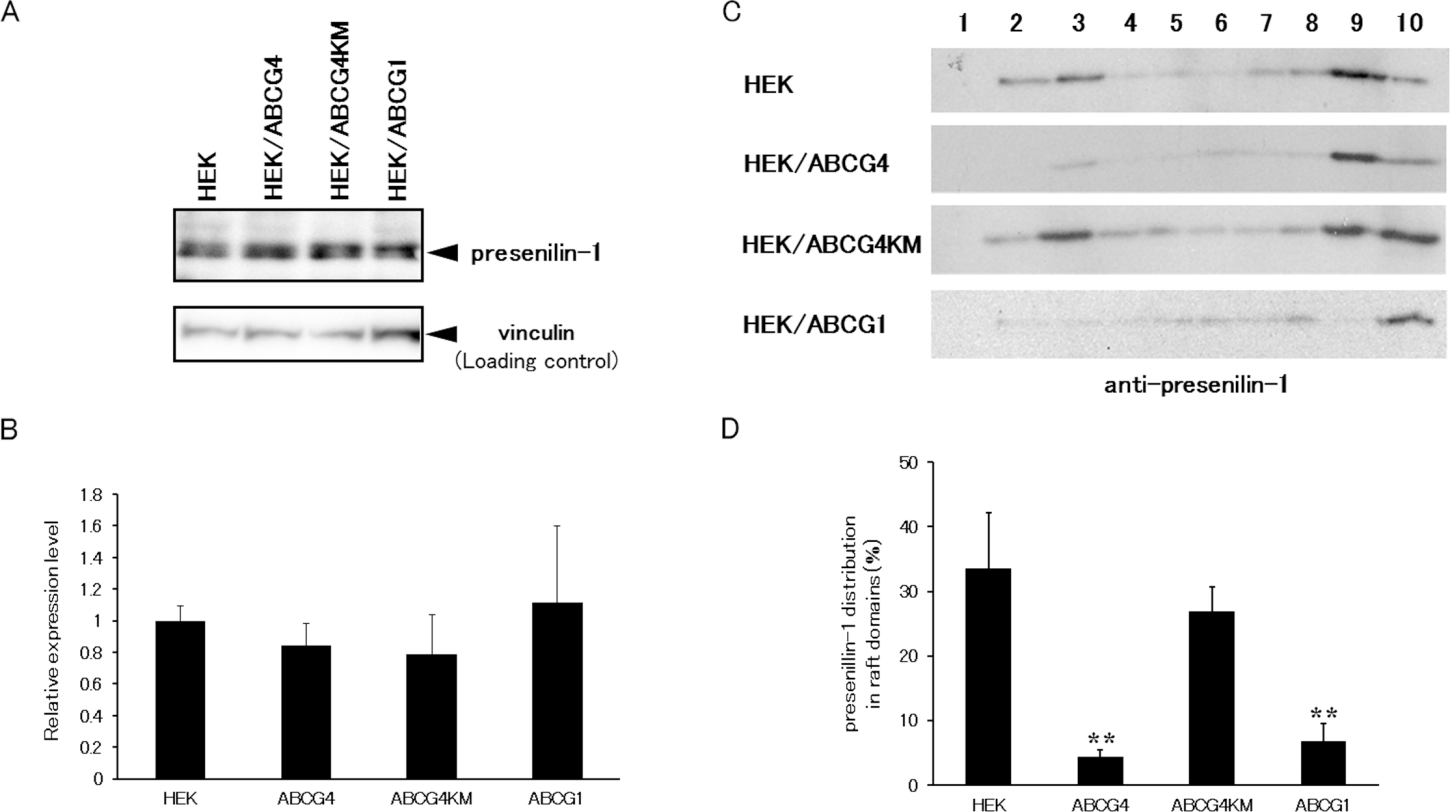
Distribution of presenilin-1 was altered by ABCG1 or ABCG4. (A) HEK293, HEK/ABCG4, HEK/ABCG4-KM, or HEK/ABCG1 cells were collected and presenilin-1 N-terminal fragment was detected by immunoblotting. (B) The amounts of presenilin-1 N-terminal fragment detected by immunoblotting were analyzed. The data represent the expression levels of presenilin-1 normalized by vinculin relative to that in HEK293 cells. Values are represented with the SD. (C) HEK293, HEK/ABCG4, HEK/ABCG4-KM, or HEK/ABCG1 cells were incubated in DMEM containing 0.02% BSA for 24 h, and treated with lysis buffer containing 1% Triton X-100 on ice. Cell lysates were separated using OptiPrep-gradient ultracentrifugation. Ten fractions from each sample were separated using 5−20% polyacrylamide gel electrophoresis, and presenilin-1 N-terminal fragment was detected by immunoblotting. (D) Presenilin-1 N-terminal fragment levels on Western blots were analyzed, and the average percentages of presenilin-1 in the raft domains (fractions 2 and 3) relative to total presenilin-1 levels (fractions 1−10) from five experiments are represented with the SD. ** *P* < 0.01 compared with HEK293 cells.

We have shown that ABCG1 and ABCG4 disturb raft structure [42]. It has been reported that γ-secretase functions in raft domains [11, 43]. We speculated that disruption of raft structure leads to decreased γ-secretase activity. To examine this, raft domains were separated using an OptiPrep gradient ultracentrifugation assay (S3 Fig). Caveolin-1, a raft marker, was distributed in raft fractions (fractions 2 and 3) in host HEK293 cells. Caveolin-1 was detected in raft fractions in HEK293 cells stably expressing ABCG4-KM (HEK/ABCG4-KM), but was hardly seen in HEK/ABCG1 and HEK/ABCG4 cells, as we reported previously [42]. Under this condition, the distribution of subunits of γ-secretase was investigated. The active γ-secretase is composed of 4 subunits: presenilin-1, nicastrin, PS-enhancer-2 (Pen-2), and anterior pharynx-defective-1 (APH-1). Among these, presenilin-1 is a catalytic subunit. Endogenous presenilin-1 was detected in HEK293 cells (Fig 5A). The expression levels of presenilin-1 were similar in HEK/ABCG4, HEK/ABCG4-KM, and HEK/ABCG1 cells (Fig 5A and 5B). Then, we examined if the distribution of presenilin-1 is affected by the expression of ABCG1 and ABCG4. In host HEK293 cells, endogenous presenilin-1 was distributed in raft fractions (fractions 2 and 3), whereas some was detected in non-raft fractions (fractions 8−10) (Fig 5C). Little presenilin-1 was observed in raft fractions from HEK/ABCG1 and HEK/ABCG4 cells, but it was detected in the non-raft fractions. In contrast, presenilin-1 was observed in raft fractions from HEK/ABCG4-KM cells. More than 30% of the presenilin-1 was localized in raft domains in HEK293 cells, but only 7% and 4% were detected in raft domains in HEK/ABCG1 and HEK/ABCG4 cells, respectively (Fig 5D). This result suggests that ABCG1 and ABCG4 reduce presenilin-1 levels in raft domains. There was no significant difference between the distributions of presenilin-1 in the raft domains of HEK293 and HEK/ABCG4-KM cells. This shows that the change in presenilin-1 localization was dependent on the ATPase activity of ABCG4. Similar results were observed for the other γ-secretase subunit nicastrin (S4 Fig). Endogenous nicastrin was detected in raft fractions from HEK293 and HEK/ABCG4-KM cells, whereas raft fractions from HEK/ABCG1 and HEK/ABCG4 cells did not contain nicastrin. These results suggest that ABCG1 and ABCG4 redistribute γ-secretase from raft domains to non-raft domains, possibly by destructing raft structures, and that the altered distribution reduces γ-secretase activity, leading to the reduced Aβ secretion.

### Suppression of ABCG1 and ABCG4 increases γ-secretase activity and Aβ levels

**Fig 6:**
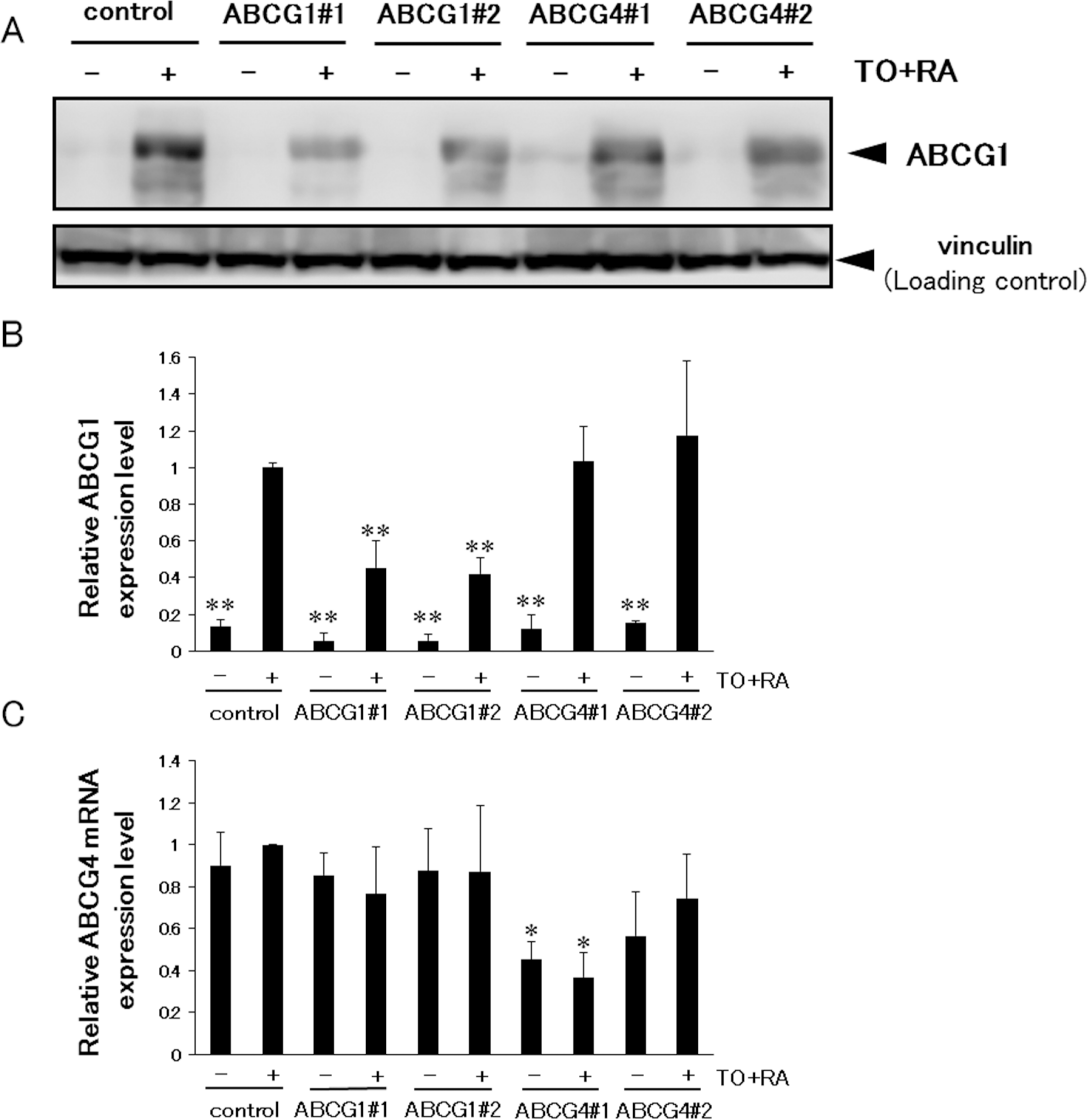
Suppression of ABCG1 and ABCG4 by siRNA. (A) SH-SY5Y cells were transfected with siRNA against ABCG1, ABCG4, or scrambled siRNA (control), and allowed to differentiate for 3 days. After 16-h incubation with or without TO901317 (TO) and 9-*cis* retinoic acid (RA), cells were collected and ABCG1 was detected by immunoblotting. (B) The amount of ABCG1 detected by immunoblotting was analyzed. The data represent the expression levels of ABCG1 normalized by vinculin relative to those in control cells incubated with TO and RA. Values are represented with the SD. (C) Total RNA was extracted from differentiated SH-SY5Y cells. Quantitative RT-PCR was performed and the ABCG4 mRNA expression level was normalized to 18S rRNA. Relative expression levels of ABCG4 mRNA in cells transfected with siRNA are represented against those in control cells treated with TO and RA. * *P* < 0.05; ** *P* < 0.01 compared with control cells incubated with TO and RA. All experiments were carried out in triplicate.

**Fig 7:**
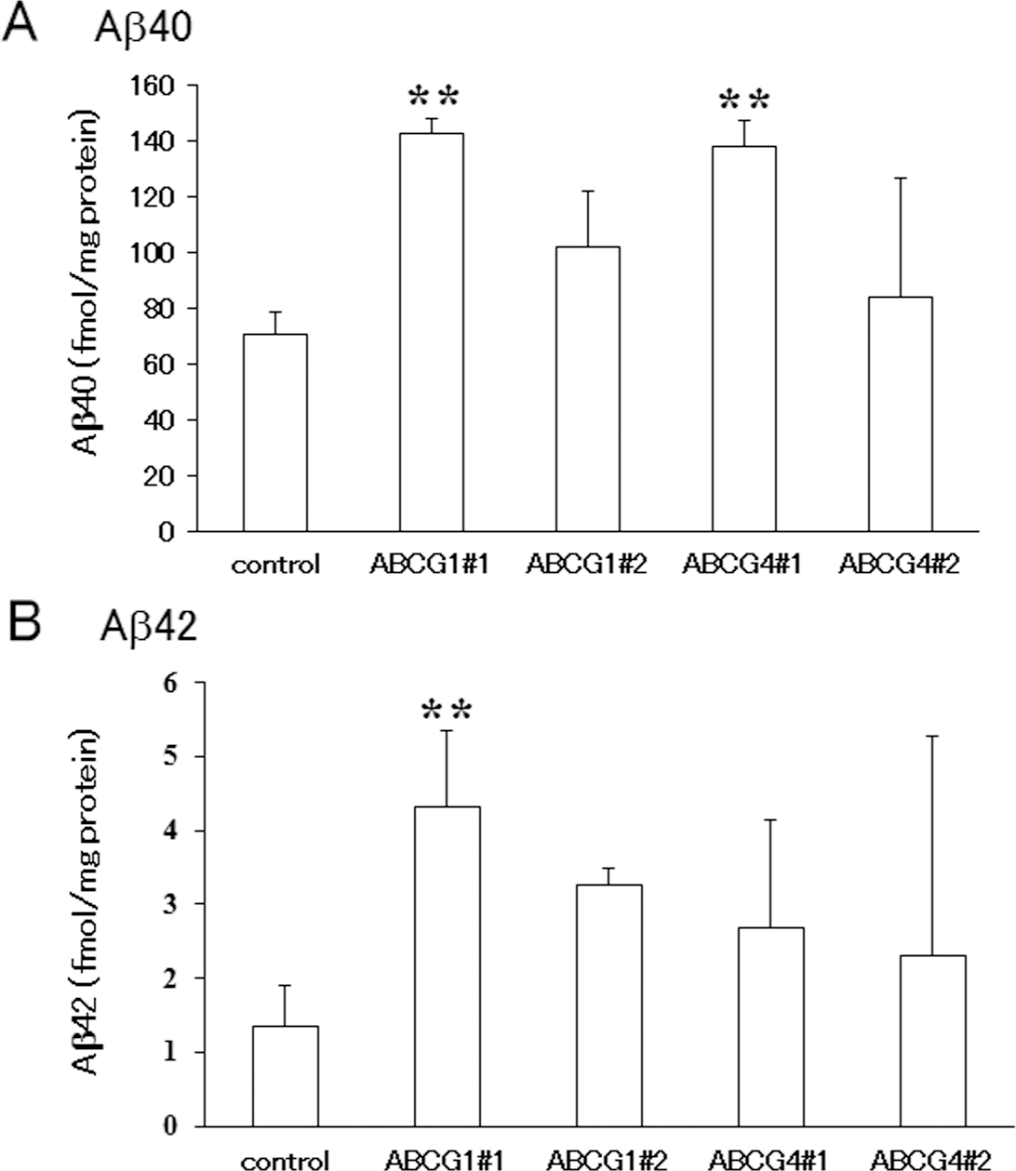
Aβ secretion from SH-SY5Y cells. SH-SY5Y cells were transfected with siRNA against ABCG1, ABCG4, or scrambled siRNA (control), and allowed to differentiate for 3 days. Cells were incubated in DMEM containing 0.02% BSA, 5 μM TO901317 and 5 μM 9-*cis* retinoic acid for 16 h. Media were collected and Aβ40 (A) and Aβ42 (B) levels were measured using an enzyme-linked immunosorbent assay. Values from three experiments were normalized based on total cellular protein, and means are represented with the SD. ** *P* < 0.01 compared with control cells.

**Fig 8:**
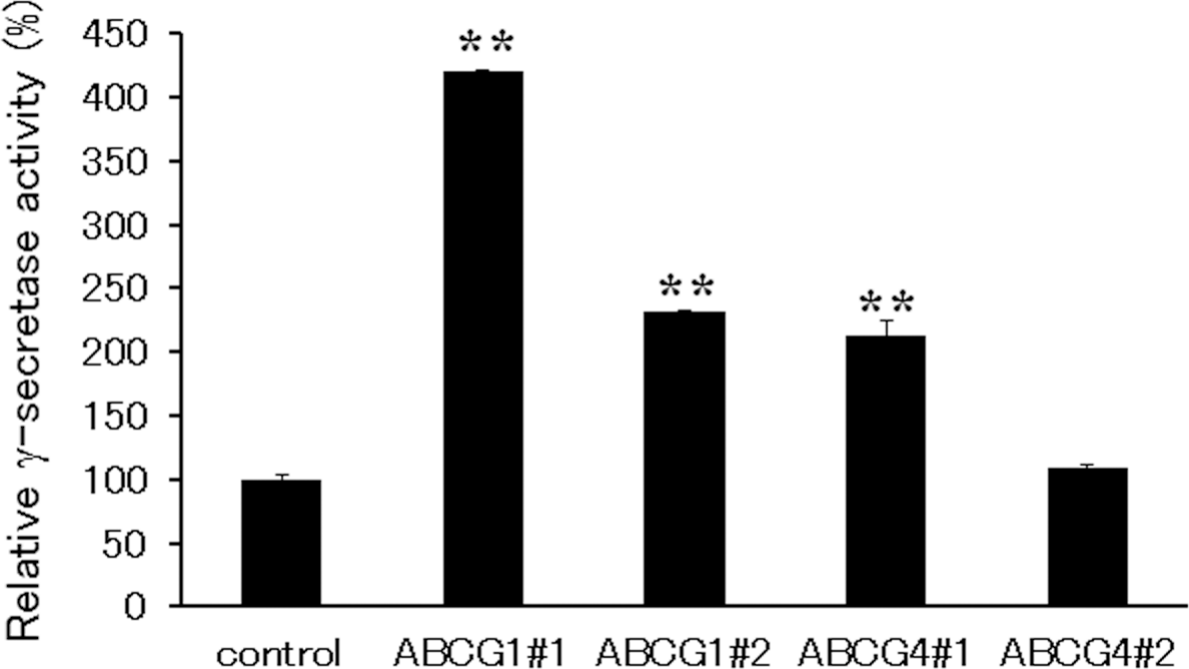
γ-secretase activity in SH-SY5Y cells. SH-SY5Y cells were transfected with siRNA against ABCG1, ABCG4, or scrambled siRNA (control), and allowed to differentiate for 3 days. Cells were incubated in DMEM containing 0.02% BSA, 5 μM TO901317, and 5 μM 9-*cis* retinoic acid for 16 h. The activity of endogenous γ-secretase was determined using a γ-secretase assay kit. Experiments were performed in triplicate and the activities relative to those observed in control cells are represented as means with the SD. ** *P* < 0.01 compared with control cells.

Because the above experiments were performed using an overexpression system, we also examined the effects of endogenous ABCG1 and ABCG4 by using SH-SY5Y cells. SH-SY5Y cells were differentiated to neuron-like cells by all-*trans* retinoic acid, and ABCG1 and ABCG4 were suppressed by siRNAs. When differentiated SH-SY5Y cells were treated with TO901317 and 9-*cis* retinoic acid, i.e., ligands for LXR and RXR, respectively, ABCG1 expression was induced (S1C Fig). The expression level of ABCG1 in SH-SY5Y cells was lower than that in HEK293 cells transiently or stably expressing ABCG1, but the amount of endogenous ABCG1 in SH-SY5Y cells treated with TO901317 and 9-*cis* retinoic acid was comparable to that in ABCG1-overexpressing HEK293 cells (S1A, S1C and S1D Fig). Treatment of cells with two siRNAs against ABCG1 (ABCG1#1 and ABCG1#2) decreased the induced ABCG1 expression levels to less than half (Fig 6A and 6B). Without induction of ABCG1, siRNA treatment also reduced the ABCG1 level as compared to that for the control. Expression levels of ABCG1 in cells treated with two siRNAs against ABCG4 (ABCG4#1 and ABCG4#2) did not change. Because an antibody that recognizes endogenous ABCG4 is not available, we analyzed mRNA levels of ABCG4 (Fig 6C). Treatment of cells with TO901317 and 9-*cis* retinoic acid did not affect ABCG4 mRNA levels. The ABCG4 mRNA level significantly decreased when using the siRNA ABCG4#1, while ABCG4#2 relatively but not significantly reduced the ABCG4 mRNA level. siRNAs against ABCG1 did not change ABCG4 mRNA levels. Under this condition, endogenous APP levels were analyzed. Expression levels of APP, however, were low and it was difficult to quantify and compare them by western blotting (data not shown). Then, the amounts of Aβ secreted from cells treated with TO901317 and 9-*cis* retinoic acid were measured in the absence of lipid acceptors (Fig 7). Aβ40 secretion increased when ABCG1 and ABCG4 were suppressed by ABCG1#1 and ABCG4#1, respectively, although it was not increased significantly by ABCG1#2 and ABCG4#2 treatment. Aβ42 secretion was increased by suppression of ABCG1 by ABCG1#1. We also measured γ-secretase activity to determine whether its alteration accounts for the altered Aβ secretion. Suppression of ABCG1 and ABCG4 increased γ-secretase activity, except in the case of ABCG4#2 (Fig 8). These results support the idea that ABCG1 and ABCG4 affect γ-secretase activity, leading to altered Aβ secretion.

### Aβ mass in cerebrospinal fluid increased in Abcg1 null mice

**Fig 9:**
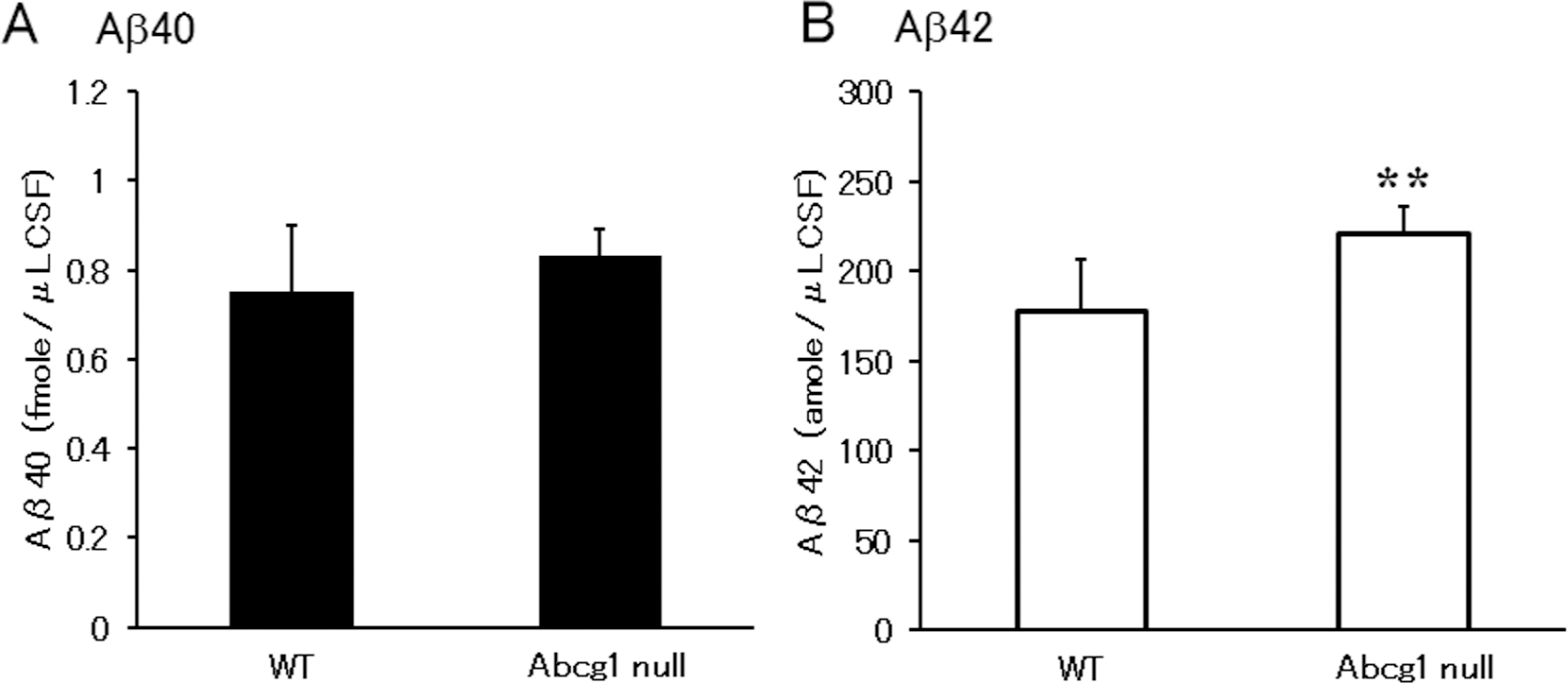
Aβ in mouse CSF increased in Abcg1 null mice. Aβ40 (A) and Aβ42 (B) levels of CSF were measured by ELISA Wako kit. Statistical significance was analyzed by Student’s t-test. ** *P* < 0.01 compared with wild type mice.

To examine the *in vivo* involvement of ABCG1 on modulating Aβ level in CSF, CSF was harvested from twelve month or older (15.42±3.95 month-old) C57BL/6 wild type and Abcg1 null mice and Aβ was measured (Fig 9). Statistically significantly higher levels of Aβ42 were observed in the Abcg1 null mice (Aβ40; 0.750±0.149 in WT vs 0.831±0.062 fmole/μL in Abcg1 null, P = 0.864, n = 8 and Aβ42; 0.177±0.0291 in WT vs 0.220±0.0149 fmole/μL in Abcg1 null, P = 0.006, n = 8), which supports the concept that reduction of ABCG1 increases production of Aβ peptides in CSF.

## Discussion

In this study, we investigated the effects of ABCG1 and ABCG4 on APP processing and γ-secretase activity. We demonstrated that ABCG4 as well as ABCG1 suppressed Aβ production and changed the localization of γ-secretase subunits from raft to non-raft domains. This is the first report that ABCG4 affects the processing of APP. Furthermore, we suggest that the altered distribution of γ-secretase causes the reduced secretion of Aβ. We also demonstrated that Aβ secretion from neuron-like cells in which ABCG1 and ABCG4 were suppressed increased and that Aβ42 in Abcg1 null mice significantly increased in CSF. These findings suggest that ABCG1 or ABCG4 can suppress Aβ production and plaque formation of Aβ.

There are conflicting reports about the effects of ABCG1 on the production of Aβ. Kim *et al*. reported that transient expression of ABCG1 in Chinese hamster ovary cells that stably expressed human APP reduced Aβ secretion without affecting cellular APP or soluble APPα levels [21]. On the other hand, Tansley *et al*. reported that transient expression of ABCG1 in HEK/APPsw cells increased the production of sAPPα, sAPPβ, CTFα, CTFβ, and Aβ, possibly due to increased levels of APP on the plasma membrane [38]. In this study, we have shown that transient expression of ABCG1 in HEK/APPsw cells increased production of APP, sAPPα, sAPPβ, CTFα, and CTFβ but decreased the secretion of Aβ. We used a similar expression system as Tansley *et al*., and our results are consistent with their study in terms of the increased production of APP, sAPPα, sAPPβ, CTFα, and CTFβ. Our result is, however, consistent with Kim’s study but not with Tansley’s study with regard to the secreted Aβ levels. The reason for this discrepancy is unclear, although differences in ABCG1 expression levels between the studies may be one of the causes of different Aβ levels. Alternatively, the culture conditions may have affected Aβ levels, because an absence of HDL suppressed Aβ levels as shown in Fig 3, suggesting that the effects of ABCG1 and ABCG4 are dependent on the presence of cholesterol acceptors and donors. The different cell lines may have resulted in different levels of APP, sAPPα, sAPPβ, CTFα, and CTFβ. If this is the case, studies using neurons are essential to understand the effects of ABCG1 on APP processing and Aβ secretion in the CNS and to reveal the relationship between the expression of ABCG1 and Alzheimer’s disease.

MβCD extracted cholesterol from cells and decreased Aβ secretion (data not shown) [5, 11, 12]. The mechanism underlying this process, however, likely differs from that involving ABCG1 and ABCG4. Expression of ABCG1 or ABCG4 increased the levels of cellular APP, sAPPα, and sAPPβ, whereas MβCD treatment did not change cellular APP levels, increased sAPPα levels, and decreased sAPPβ levels (data not shown). We suggest that the effects of MβCD were a consequence of removing cholesterol from the plasma membrane, and that the effects of ABCG1 and ABCG4 were caused by cholesterol redistribution in the plasma membrane because Aβ secretion was suppressed even under the condition without lipid acceptors, in which ABCG1- or ABCG4-mediated lipid efflux did not occur. Kim *et al*. also showed that Aβ generation was suppressed by ABCG1 even in the absence of lipid acceptors, such as apoE-disc [21]. These results suggest that suppressed Aβ secretion is not dependent on the lipid efflux by ABCG1 or ABCG4. Suppressed Aβ secretion was not observed in the cells expressing ABCG4-KM, suggesting that the ATPase activity of ABCG4 was required for the suppression. Therefore, ABCG1- and ABCG4-mediated lipid redistribution may lead to decreased secretion of Aβ.

Previous reports showed that ABCA1 expression suppressed the secretion of Aβ even in the absence of apoA-I [30, 31]; one report, however, demonstrated that ABCA1 increased Aβ secretion [36]. In the current study, we showed that both ABCG1 and ABCG4 reduced Aβ secretion, which was independent of lipid efflux. Thus, ABCA1, ABCG1, and ABCG4 may suppress Aβ secretion via a common mechanism. We demonstrated that the distributions of γ-secretase subunits on the plasma membrane changed and γ-secretase activity was reduced in the presence of ABCG1 or ABCG4. It has been reported that ABCA1 and ABCG1 redistributed membrane lipids and increased the amount of cholesterol accessible to cholesterol oxidase [41, 53], suggesting that ABCA1 and ABCG1 disrupt the raft domains, and consequently increased the area of non-raft domains. ABCG1 has been reported to reduce the sizes of lipid rafts [54]. Furthermore, we have demonstrated that ABCG1 and ABCG4 disturb raft domains [42]. Disruption of raft domains by ABCG1 and ABCG4 may disturb the localization of γ-secretase in the raft domains and reduce γ-secretase activity. This model is supported by data showing that MβCD treatment, which reduces the areas of raft domains, also decreased γ-secretase levels in the raft domains and γ-secretase activity [11, 43]. Therefore, ABCA1, ABCG1, and ABCG4 may modulate raft domain structures, leading to reduced γ-secretase activity and Aβ secretion. Aβ secretion was suppressed both in the presence or absence of HDL. Surprisingly, HDL slightly suppressed effects of ABCG1 and ABCG4 on Aβ secretion rather than enhance them. HDL may suppress disruption of raft domains by exchange of cholesterol between HDL and membranes. These suggest that lipid redistribution but not lipid efflux is responsible for modulation of raft domains and reduced Aβ secretion. Furthermore, ABCA1, ABCG1, and ABCG4 may suppress Aβ aggregation because aggregation of Aβ occurs in the raft domains [13].

Because less Aβ was secreted irrespective of increased levels of the γ-secretase substrate CTFβ, it is likely that reduced γ-secretase activity is responsible for the decreased Aβ secretion. We, however, cannot exclude the possibility that ABCG1 and ABCG4 affect the activities of α-secretase and β-secretase in addition to γ-secretase. Because β-secretase has been reported to reside in the raft domains [55], β-secretase activity may be also modulated by ABCG1 and ABCG4. Further studies to clarify the effects of ABCG1 and ABCG4 on overall APP processing are needed.

Cellular APP levels and especially surface APP levels were increased by the expression of ABCG1 and ABCG4. This is not because transcription of APP was induced, because mRNA level of APP was not altered by ABCG1 or ABCG4 (S5 Fig). APP may accumulate in cells because γ-secretase activity was reduced by ABCG1 and ABCG4. The rate of cellular APP degradation was slightly reduced by ABCG1 or ABCG4 (S2 Fig). Furthermore, endocytosis of APP may be suppressed because inhibition of endocytosis increased the amounts of cell surface APP [51]. Suppressed endocytosis may be one factor that reduces Aβ production because endocytosis is essential for β-cleavage [12]. Expression of ABCG1 and ABCG4 may alter membrane trafficking via lipid redistribution. Presenilin-1 levels were not affected by expression of ABCG1 and ABCG4, while APP levels increased in cells expressing ABCG1 and ABCG4. Disturbed raft domains by ABCG1 and ABCG4 may affect APP and presenilin-1 levels differently, because APP and presenilin-1 localize to distinct membrane domains [56]. Alternatively, APP degradation is suppressed but presenilin-1 degradation may not be affected by expression of ABCG1 and ABCG4.

Aβ40 secretion increased when endogenous ABCG1 and ABCG4 were suppressed in differentiated SH-SY5Y cells. Aβ42 secretion was also increased by suppression of ABCG1 but the increase was not significant in the case of suppression of ABCG4. ABCA1 expression might be elevated to compensate for the reduced expression of ABCG1 and ABCG4, which weakened the effects of knockdown. Aβ secretion from SH-SY5Y cells and γ-secretase activity increased when ABCG1 and ABCG4 were suppressed, suggesting that ABCG1 and ABCG4 reduce γ-secretase activity and Aβ secretion and are physiologically relevant for regulation of Aβ levels.

Aβ42 in Abcg1 null mice was significantly increased in CSF in this study. The level of this peptide in CSF has been shown to be a relatively good marker for progression of Alzheimer’s disease. The experimental animals used in this study were older than one-year (15.4±3.95 month), which is approximately half of their life span. The previous report [39], using relatively younger mice, evaluated Aβ from hippocampi tissue which presumably include CSF, interstitial fluid [57], and cytosol of neuronal and glial cells. Aβ level in CSF from this mouse model system was not indicated. The brain system is highly evolved in human compared to other organs such as liver cells or muscle cells. Thus, there are limitation using model mice to anticipate human neuronal diseases while at least one of the inter brain fluid system, CSF, indicated significantly increased Aβ level by the raft condition modified by ABCG1. Alzheimer’s disease is also known to occur at a higher incidence in females [58], so in the future, it will be important to investigate the presence of any gender differences in the accumulation of Aβ42 in Abcg1 null mice.

In summary, we have shown that the ABCG1 and ABCG4 but not ABCG4-KM affected APP processing. We demonstrated that expression of ABCG1 and ABCG4 relocated γ-secretase and reduced its activity, which led to the reduced Aβ secretion. Furthermore, we showed that Aβ secretion from neuron-like cells increased when ABCG1 and ABCG4 were suppressed and that Aβ42 level in CSF significantly increased in Abcg1 null mice compared to the wild type mice. ABCG1 and ABCG4 may play important roles in suppression of Aβ generation and pathogenesis of Alzheimer’s disease. ABCG1 and ABCG4 could be new targets for prevention and treatment of Alzheimer’s disease. Inducing expression or activity of ABCG1 and ABCG4 in the CNS may prevent amyloid plaque formation by reducing Aβ production.

## Supporting Information

S1 Fig Expression of ABCG1, ABCG4, and sodium potassium ATPase in HEK293 and SH-SY5Y cells. (A) HEK/APPsw cells were transiently transfected with ABCG4, ABCG4-KM, or ABCG1, or mock-transfected. Twenty-four hours after transfection, cells were collected and ABCG1, ABCG4, and sodium potassium ATPase (Na, K-ATPase) alpha1 subunit were detected by immunoblotting. (B) The amounts of Na, K-ATPase alpha1 subunit detected by immunoblotting were analyzed. The data represent the expression levels of Na, K-ATPase alpha1 subunit normalized by vinculin relative to that in mock-transfected cells. Values are represented with the SD. (C) SH-SY5Y cells were allowed to differentiate for 3 days. After 16-h incubation with or without TO901317 (TO) and 9-*cis* retinoic acid (RA), cells were collected and ABCG1, ABCG4, and Na, K-ATPase alpha1 subunit were detected by immunoblotting. (D) HEK293, HEK/ABCG4, HEK/ABCG4-KM, or HEK/ABCG1 cells were collected and ABCG1, ABCG4, and Na, K-ATPase alpha1 subunit were detected by immunoblotting.
(TIF)

S2 Fig Half-life of APP was extended by ABCG1 or ABCG4. (A) HEK/APPsw cells were transiently transfected with ABCG1 or ABCG4, or mock-transfected. Twenty-four hours after transfection, 100 μg/ml cycloheximide was added to the medium; cells were collected after 0, 0.5, 1, 2, and 4 h; and cellular APP was detected by immunoblotting. (B) Mature and immature APP levels on Western blots were analyzed, and the average percentages of remaining APP relative to APP levels just before adding cycloheximide are represented with the SD.
(TIF)

S3 Fig Distribution of caveolin-1 was altered by ABCG1 or ABCG4. (A) HEK293, HEK/ABCG4, HEK/ABCG4-KM, or HEK/ABCG1 cells were incubated in DMEM containing 0.02% BSA for 24 h, and treated with lysis buffer containing 1% Triton X-100 on ice. Cell lysates were separated using OptiPrep-gradient ultracentrifugation. Ten fractions from each sample were separated using 5−20% polyacrylamide gel electrophoresis, and caveolin-1 was detected by immunoblotting.
(TIF)

S4 Fig Distribution of nicastrin was altered by ABCG1 or ABCG4. HEK293, HEK/ABCG4, HEK/ABCG4-KM, or HEK/ABCG1 cells were incubated in DMEM containing 0.02% BSA for 24 h, and treated with lysis buffer containing 1% Triton X-100 on ice. Cell lysates were separated using OptiPrep-gradient ultracentrifugation. Ten fractions from each sample were separated using 5–20% polyacrylamide gel electrophoresis, and nicastrin was detected by immunoblotting.
(TIF)

S5 Fig APP mRNA level was not changed by ABCG1 or ABCG4. Total RNA was extracted from HEK293, HEK/ABCG4, HEK/ABCG4-KM, or HEK/ABCG1 cells. Quantitative RT-PCR was performed and APP mRNA expression level was normalized to 18S rRNA. Relative expression levels of APP mRNA in HEK/ABCG4, HEK/ABCG4-KM, or HEK/ABCG1 cells were represented against that in host HEK293 cells.
(TIF)

## Abbreviations

ABC: ATP-binding cassette
Aβ: amyloid beta
apo: apolipoprotein
APP: amyloid precursor protein
BSA: bovine serum albumin
CNS: central nervous system
CSF: cerebrospinal fluid
CTF: carboxy-terminal fragment
DMEM: Dulbecco’s modified Eagle’s medium
HEK: human embryonic kidney
HDL: high-density lipoprotein
LpE: apoE-containing lipoprotein
LXR: liver X receptor
MβCD: methyl-β-cyclodextrin
RXR: retinoid X receptor
sAPP: secreted APP
