# Supplementary figures and images for: ABCG1 and ABCG4 Suppress γ-Secretase Activity and Amyloid β Production
Source: PLoS One. 2016 May 19;11(5):e0155400. doi: 10.1371/journal.pone.0155400 (PMC4872999; doi:10.1371/journal.pone.0155400)

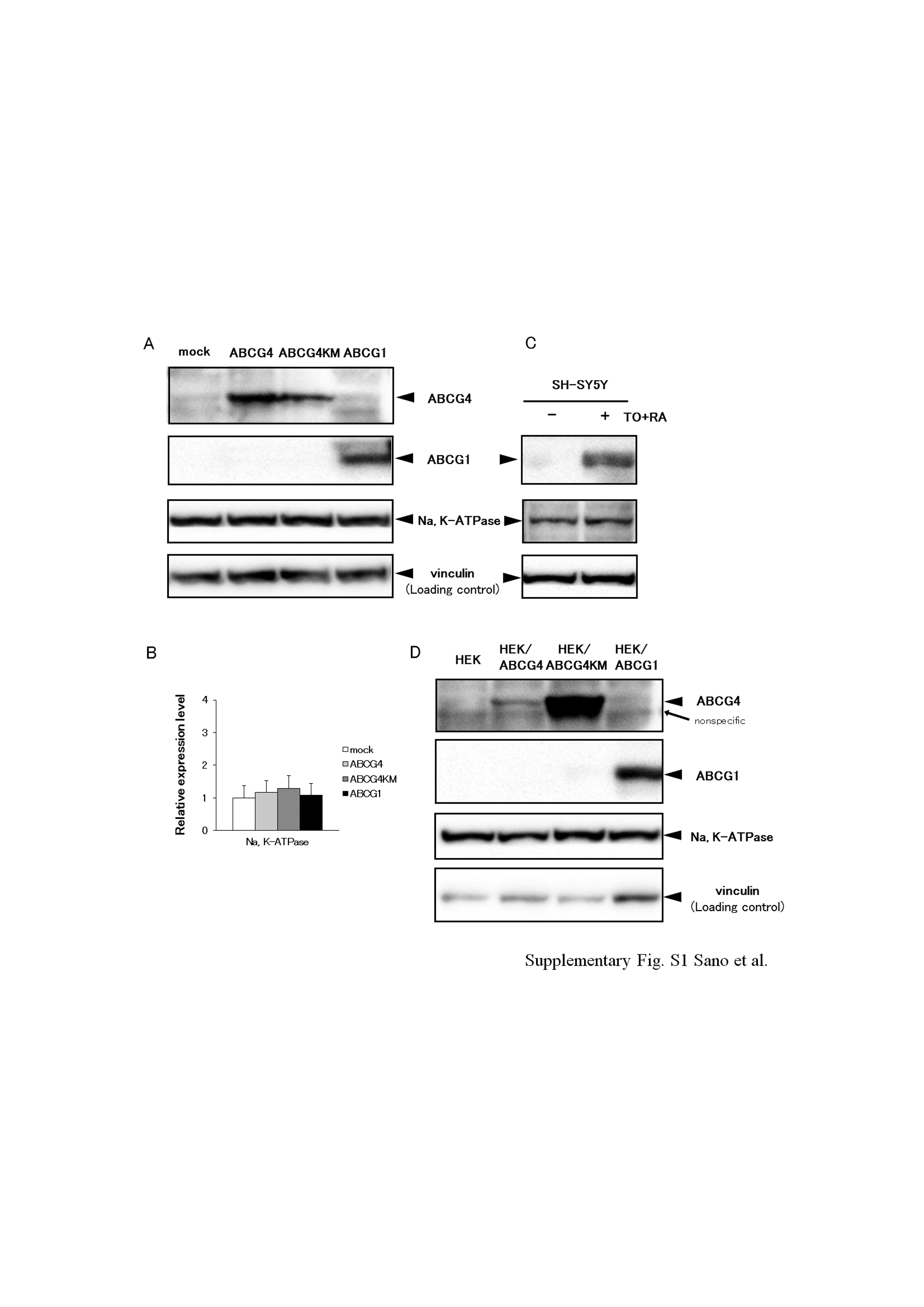

Supplement: S1 Fig — (A) HEK/APPsw cells were transiently transfected with ABCG4, ABCG4-KM, or ABCG1, or mock-transfected. Twenty-four hours after transfection, cells were collected and ABCG1, ABCG4, and sodium potassium ATPase (Na, K-ATPase) alpha1 subunit were detected by immunoblotting. (B) The amounts of Na, K-ATPase alpha1 subunit detected by immunoblotting were analyzed. The data represent the expression levels of Na, K-ATPase alpha1 subunit normalized by vinculin relative to that in mock-transfected cells. Values are represented with the SD. (C) SH-SY5Y cells were allowed to differentiate for 3 days. After 16-h incubation with or without TO901317 (TO) and 9-cis retinoic acid (RA), cells were collected and ABCG1, ABCG4, and Na, K-ATPase alpha1 subunit were detected by immunoblotting. (D) HEK293, HEK/ABCG4, HEK/ABCG4-KM, or HEK/ABCG1 cells were collected and ABCG1, ABCG4, and Na, K-ATPase alpha1 subunit were detected by immunoblotting. (TIF) [file pone.0155400.s001.tif]

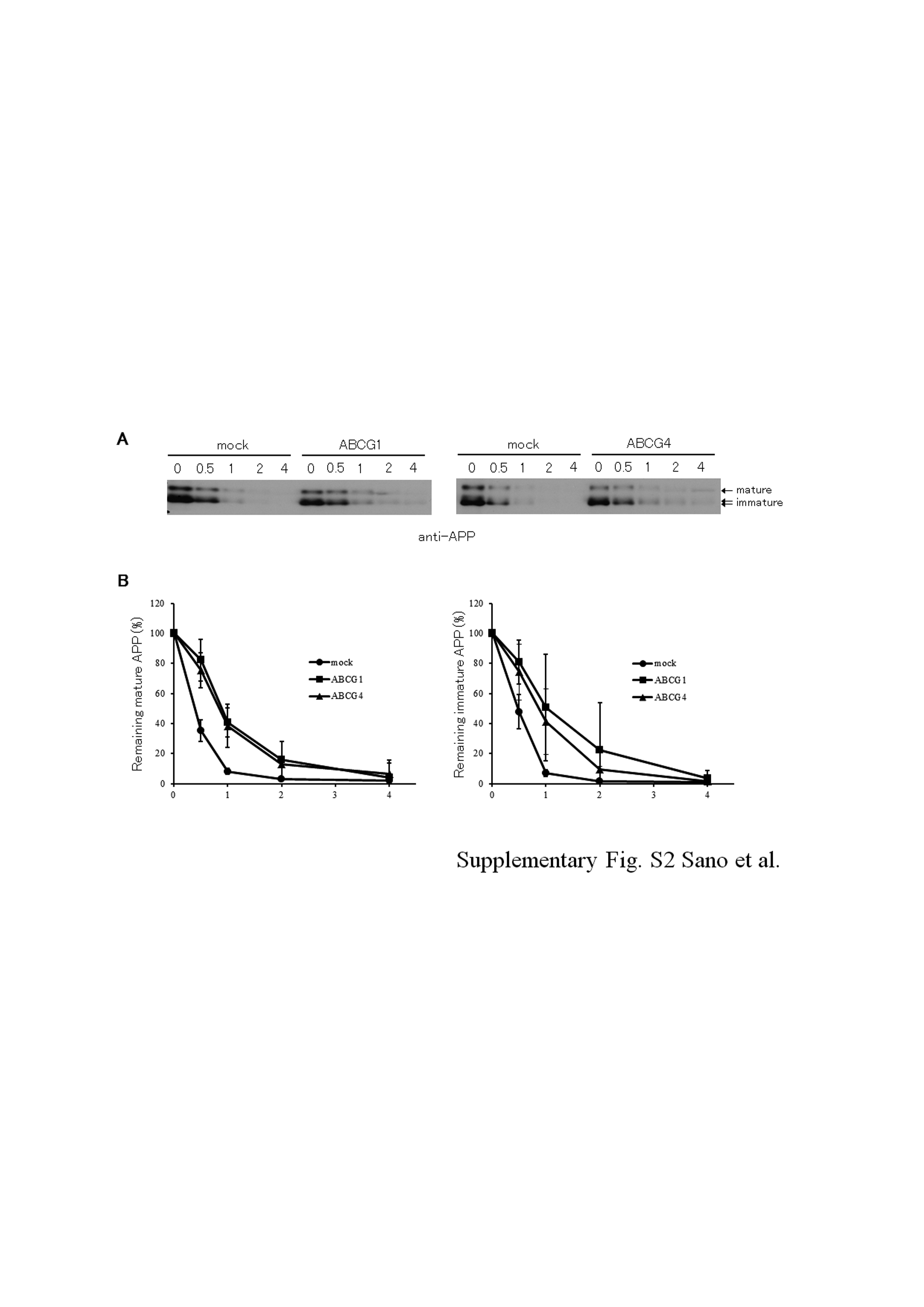

Supplement: S2 Fig — (A) HEK/APPsw cells were transiently transfected with ABCG1 or ABCG4, or mock-transfected. Twenty-four hours after transfection, 100 μg/ml cycloheximide was added to the medium; cells were collected after 0, 0.5, 1, 2, and 4 h; and cellular APP was detected by immunoblotting. (B) Mature and immature APP levels on Western blots were analyzed, and the average percentages of remaining APP relative to APP levels just before adding cycloheximide are represented with the SD. (TIF) [file pone.0155400.s002.tif]

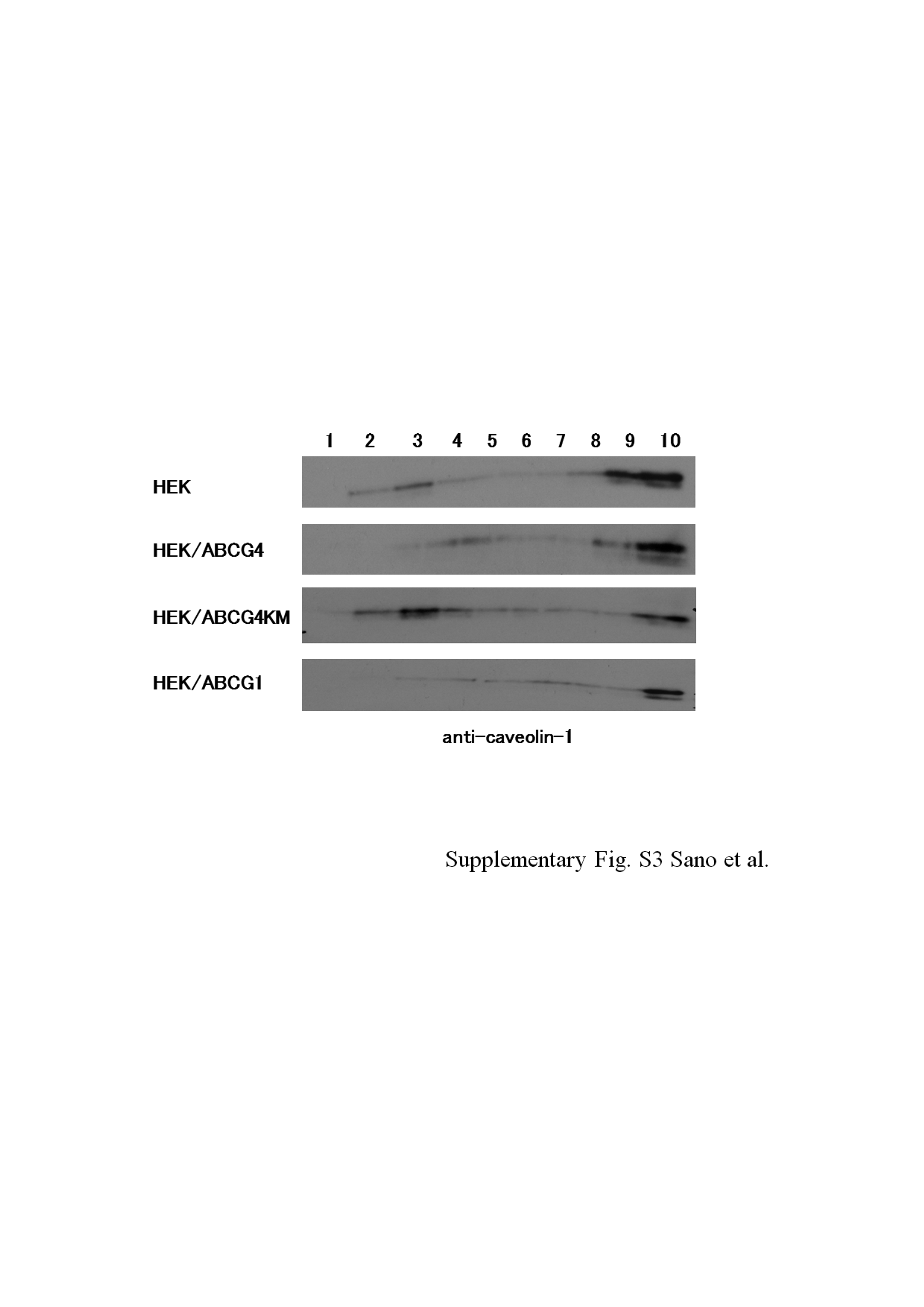

Supplement: S3 Fig — (A) HEK293, HEK/ABCG4, HEK/ABCG4-KM, or HEK/ABCG1 cells were incubated in DMEM containing 0.02% BSA for 24 h, and treated with lysis buffer containing 1% Triton X-100 on ice. Cell lysates were separated using OptiPrep-gradient ultracentrifugation. Ten fractions from each sample were separated using 5−20% polyacrylamide gel electrophoresis, and caveolin-1 was detected by immunoblotting. (TIF) [file pone.0155400.s003.tif]

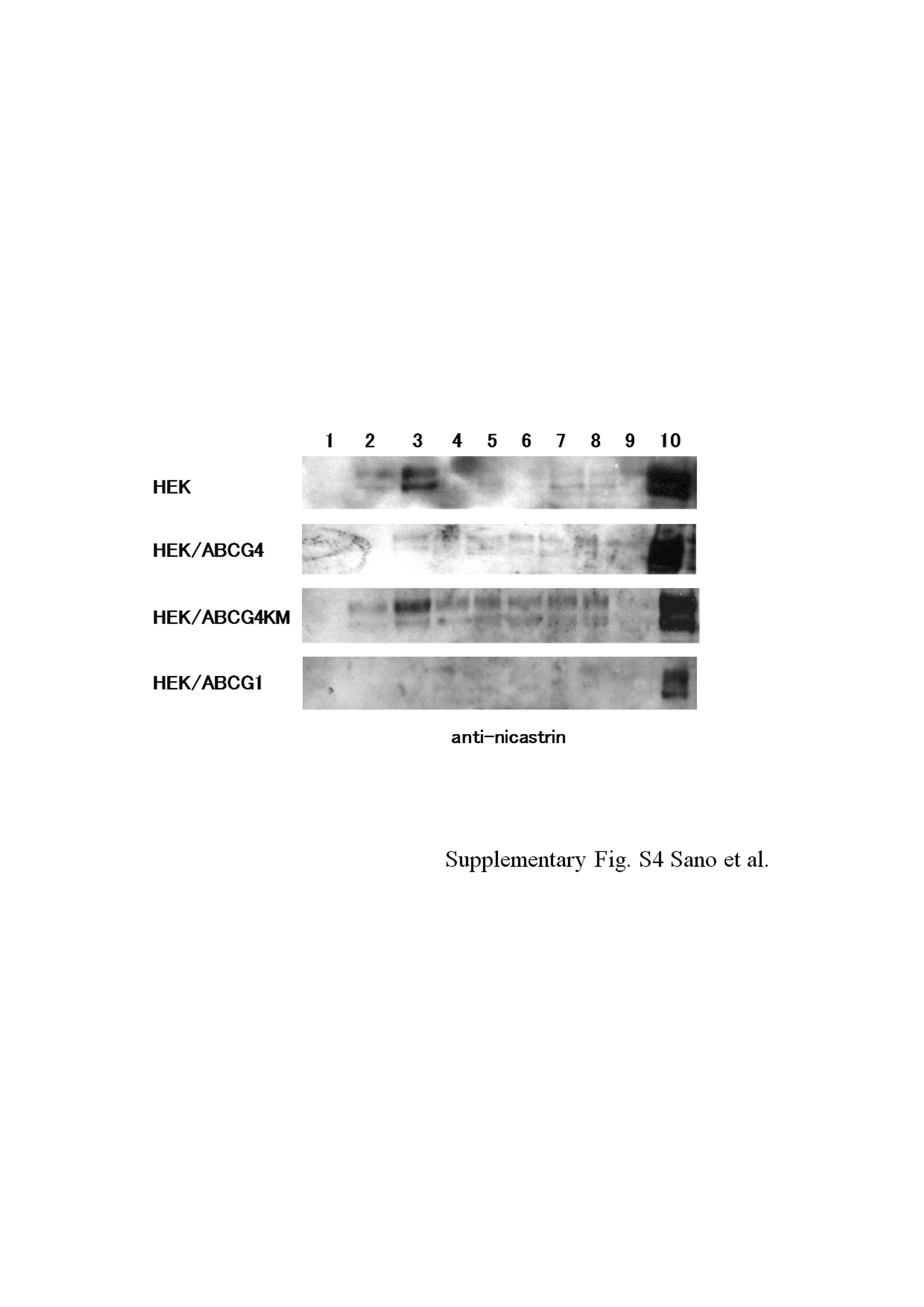

Supplement: S4 Fig — HEK293, HEK/ABCG4, HEK/ABCG4-KM, or HEK/ABCG1 cells were incubated in DMEM containing 0.02% BSA for 24 h, and treated with lysis buffer containing 1% Triton X-100 on ice. Cell lysates were separated using OptiPrep-gradient ultracentrifugation. Ten fractions from each sample were separated using 5–20% polyacrylamide gel electrophoresis, and nicastrin was detected by immunoblotting. (TIF) [file pone.0155400.s004.tif]

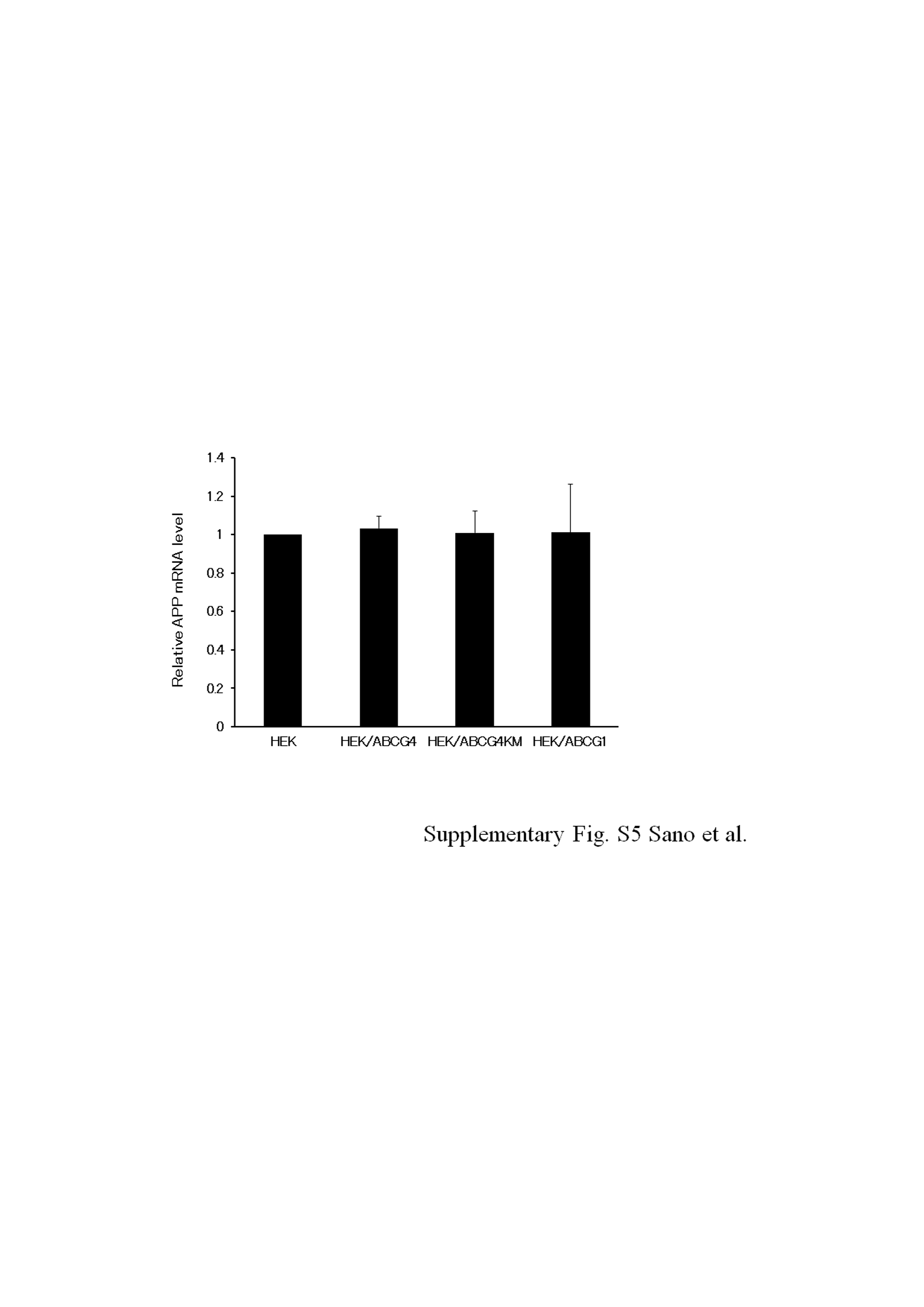

Supplement: S5 Fig — Total RNA was extracted from HEK293, HEK/ABCG4, HEK/ABCG4-KM, or HEK/ABCG1 cells. Quantitative RT-PCR was performed and APP mRNA expression level was normalized to 18S rRNA. Relative expression levels of APP mRNA in HEK/ABCG4, HEK/ABCG4-KM, or HEK/ABCG1 cells were represented against that in host HEK293 cells. (TIF) [file pone.0155400.s005.tif]
